# Feasibility study of the implementation of health promoting processes in a secondary school and ways to capture its impact on adolescent lifestyle choices^☆^

**DOI:** 10.1016/j.puhip.2025.100591

**Published:** 2025-02-15

**Authors:** C.A. Forbes, A.J. Williams, K. Wyatt

**Affiliations:** a University of Exeter, Exeter, United Kingdom; b University of Edinburgh, Edinburgh, United Kingdom

**Keywords:** Adolescence, Health promotion, Complex adaptive systems, Schools

## Abstract

**Objectives:**

Schools are environments that influence adolescent health choices; understanding schools as complex adaptive systems, we have developed a series of processes that are adaptive to the school context, to support schools to create the conditions for health promotion. The aim of this study was to determine the feasibility and acceptability of capturing the impact of implementing the health promoting school (HPS) process.

**Study design:**

feasibility study.

**Methods:**

A purposefully selected secondary school in England, with high Free School Meal (FSM) allocation, was recruited to implement the HPS processes, which includes an annual school audit. We developed a questionnaire, to capture lifestyle behaviours and school culture, completed before the audit and 9 months after. Descriptive analysis analysed the questionnaire responses to understand whether it captured similar responses to the audit. Post intervention interviews with staff and a focus group with students assessed the acceptability and practicality of the intervention and study design.

**Results:**

The HPS processes were implemented September 2022–September 2023. Students (n = 337), families (n = 49) and staff (n = 22) completed the school audit in November 2022 and November 2023. 237 students completed The Lifestyle and School Questionnaire at baseline (September 2022) and 210 at follow-up (June 2022). Following the initial school audit, the need to improve the school food was identified and became the school focus; results from the second audit reflected a small positive shift in students’ opinion of food provision. It was feasible to capture lifestyle and school culture data using the questionnaire and the same food related priority was captured by the initial questionnaire. However, the timing of the implementation of the changes to the school context meant that this was not captured in the follow up questionnaire results.

**Conclusion:**

This study demonstrates that it is possible to administer questionnaires regarding adolescent lifestyle choices in schools. These findings also suggest that it is feasible and acceptable to implement a set of HPS processes. More research is needed to demonstrate an impact on individual health behaviours.

What this study adds to the existing evidence base and implications for policy and practice,

• The HPS Process is feasible and acceptable and led to changes in the school food provision
• School staff, students and their families all identified school food as a barrier to their health.
• We developed a questionnaire to capture the impact of the changes on school culture and student health and wellbeing which was feasible and acceptable to administer and captured the same issues as the HPS audit
• While the HPS process helps schools identify and take appropriate action, involving wider school stakeholders and capturing the change in health and culture remain challenging

## 1 Introduction

Schools are recognised as settings that influence adolescent health choices; however, they face significant barriers in creating a culture that promotes healthy lifestyle choices [3,4]. Leading a healthy lifestyle is important for future adolescent health outcomes. Currently in England, there is no national government policy or assessment criteria for health promotion in schools, although there is a ‘Healthy Schools Rating Scheme’, which is a free voluntary self-assessment tool. The majority of schools in England have converted to ‘academies’,^1^ which gives schools more control over their spending than traditional grant maintained schools and gives local authorities less say. This creates a context in which the motivations and attitudes of Head Teachers and Business Managers towards supporting healthy behaviours becomes crucial as to whether a health-promoting school culture can successfully be created [5].

School-based diet and physical activity public health research to date has largely focused on interventions that are aimed at changing individual health behaviours rather than focusing on the school context and creating a healthy school environment. However, whole school approaches to health promotion, such as The World Health Organization (WHO) Health Promoting Schools (HPS) framework, demonstrate that they are effective in supporting healthy lifestyle behaviours [6,7]. Incorporating health-promotion into a school's core values and beliefs requires system level change. Schools are complex adaptive systems, suggesting that programmes to support health and wellbeing should be conceptualised as a series of processes, which are adaptive to the local school context and start with a whole school approach to identifying the issues for that particular school [8]. Public health problems that emerge as a property of a complex system cannot necessarily be solved with a simple, single intervention, but the interacting factors within the system that can potentially be reshaped to generate a more desirable set of outcomes [9]. Creating the conditions for complex systems to change, necessitates the system needing to recognise the problem [10]. Previous research by the authors created a set of processes for school staff, students and families to identify and respond to the barriers to health in their school; we have reported on piloting the process in secondary schools [11].

The new MRC framework for developing and evaluating complex interventions recommends a systems approach to development and evaluation, recognising the importance of how complex interventions interact in a particular context [12]. How we evaluate complex systems interventions is a challenge [13,14], the guidance suggests that there is a need to understand on how an intervention, or process, is embedded into a given setting and how emergent outcomes can be captured [9]. Capturing the impact of complex interventions is about understanding how the activity contributes to reshaping a system rather than focusing solely on individual behaviour. One of the challenges for the evaluation of complex system interventions in schools is deciding what outcomes to measure, how best to capture system level change and whether there are individual measures, which can capture changes in school ethos and values. A further consideration for any evaluation is the burden it places on participants and what can be captured which would help understand what changes have been made. The aim of this study was to determine the feasibility and acceptability of the HPS process and how to capture its impact on school culture and individual behaviours.

## 2 Methods

### 2.1 Study design and development

**Fig. 1:**
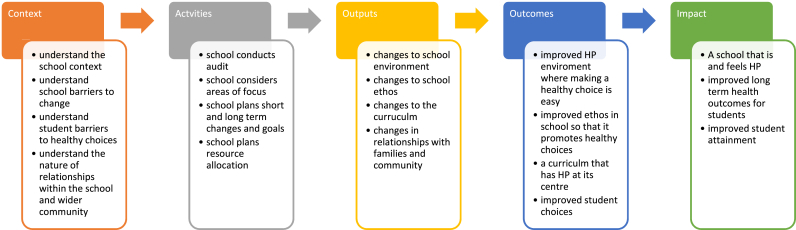
Theory of change.

This feasibility and acceptability study sought to capture both the implementation process and its impact on the school system. We developed a questionnaire, based on our theory of change (Fig. 1) to ascertain whether it captured changes in behaviour and perception of school culture and hence acted as an evaluative research tool alongside the HPS process [11].

#### 2.1.1 Public involvement and engagement (PIE)

This project had a Young Person's Advisory Group to guide the work of the project; it consisted of 8–10, 12–16 years olds of mixed genders from a similar school to those in this study. They helped design and pilot the Lifestyle and School Questionnaire, advised on the student information sheets and consent forms. This led to changes in wording and format to make it as clear and low burdensome as possible for participants to complete.

#### 2.1.2 HPS process development

Schools are described as ‘social complex adaptive systems’ making it hard to predict whether and how schools will respond to and sustain programmes designed externally to alter the school ‘system’ [15]. Understanding schools as complex adaptive systems suggests that changes to the system come about because of sense making across the system and creating feedback loops, which allow the system to self-organise; rather than the application of an outside programme [15,16]. A core concept for understanding the adoption and sustainability of system change is recognising and understanding the local context. Fig. 1 is the theory of change for the HPS process.

The aim of the HPS processes is to identify the context specific nature of the barriers in secondary schools to healthy lifestyles so that schools can make changes knowing they are responding to issues staff, students and their families have identified; this in turn leads to changes to the wider environment and a shift towards a health promoting culture, which, over time, leads to changes in lifestyle choices of adolescents. The process includes an audit that allows schools to take a lens to their context and identify the barriers and facilitators to physical and mental wellbeing [Appendix 1]. Schools are provided with a set of accessible, easy to use, up-to-date resources that support them to reflect on initiatives and activities to support their goals and create a health promoting school. A central tenet of the process is that schools take ownership of the process and repeat the process annually to identify and respond to the emergent barriers to health.

#### 2.1.3 Context

The setting was a purposely recruited secondary school in England. Inclusion criteria were that they were located in an area of economic deprivation determined by Free School Meal (FSM)^2^ allocation and provided education for students aged 11–16 years. The school was selected for capacity to work with us, commitment to engage with the processes and readiness to make changes to the school environment.

#### 2.1.4 Participants

All students in the secondary school were eligible to take part in the HPS process and all the students aged 11–15 years were recruited to complete the questionnaire.^3^ Informed consent was sought from students; families were informed of the study and an opt-out consent procedure was used as the research was considered low risk and students were deemed able to consent themselves.

Interviews were conducted with school staff who had been involved in the implementation of the process who consented to take part. A mixed age focused discussion with students from the school's student parliament, was also conducted after the implementation of the HPS process and follow-up questionnaire capture. Questions focused on their awareness of the HPS process, their opinion of it and aspects they would change.

#### 2.1.5 Process delivery

The school implemented the HPS processes during one academic year (September 2022–September 2023) and circulated the audit [Appendix 1] within the school and to parents/carers. The main researcher worked with the school to guide the implementation, analyse the findings from the audit and support the school as they considered the findings and decided what changes to be made.

### 2.2 Data collection

Feasibility and acceptability of the HPS process were assessed through the audit participation rates, usability of the data to the school and the interviews. Feasibility of the evaluation process was assessed through questionnaire participation, and the coherence between the questionnaire and audit findings.

Sanson-Fisher et al. state three key questions are being addressed in any evaluation of systems-oriented public health interventions,

a) ‘*Has a change occurred*
b) Did the change occur as a result of the intervention and not some extraneous factor or cause, and
c) *Is the degree of change perceived to be significant or important to stakeholders*?’ [17](p13)

With our complexity-informed HPS approach, the primary outcomes cannot be predicted in advance of the audit where the school identifies the barriers to health and selects their specific focus. Subsequently we needed an evaluation approach that could capture change in processes and outcomes, while not placing unnecessary burden on the school or student. Therefore, there were two key aspects to determining the feasibility of the evaluation approach,

• Can data be collected on a variety of potentially impacted outcomes in a way that a change would be detected?
• Is the format of data collection feasible and acceptable to the school and students?

#### 2.2.1 Questionnaire data

In discussion with schools, a questionnaire, administered by the school, was felt to be the most feasible and acceptable process for trying to capture change. However, there are a lack of measures to capture the health culture of a school (at a system level) as well as healthy lifestyle behaviours of the students [18]; we therefore developed a questionnaire, the Lifestyle and School Questionnaire, based on our theory of change [Appendix 2]. The questionnaire was developed to be very low burden for schools to administer and students to complete. Where possible we selected questions from established questionnaires (Adolescent Lifestyle questionnaire [19], CYRM-28 [20], UN guidelines [21] and School Climate Questionnaire [22]) with an additional five questions developed by the authors to capture an overarching view of school culture. Capturing long-term data in schools is a challenge especially due to a year group leaving every year, therefore the aim of this questionnaire was to not necessarily capture individual changes but rather a shift of response in the whole cohort [18,23]. Students completed the online questionnaire in tutor class on tablets, administered by teachers.

The questionnaire [appendix 2] included 37 questions across four domains: physical activity (5), diet (9), wellbeing (13) and school (10). Each of the physical activity and diet questions related to specific behaviours and had four response options. A 5-item Likert scale was used for the wellbeing and school questions, which were about feelings and attitudes.

##### 2.2.1.1 Analysis

As this is a feasibility study, descriptive statistical methods were used to analyse the questionnaire responses and a narrative description of a comparison between the HPS audit results and questionnaire responses is provided. All the questions were scored so that more points were gained for the healthier response. Upon review, it was decided to exclude four of the questions either because they were not specific to the school context (for example, ‘Did you have breakfast before coming to school’? and ‘I know where to go in my community to get help’) or because determining the healthier response was difficult (‘Did you have school lunch’? and ‘Did you bring your own lunch to school’?)Subsequently, the scores within each of the domains were added up and converted into percentages to put each domain on a standardised scale. Finally, the individual percentages were examined and plotted in histograms before being summarised using appropriate measures of central tendency and spread to identify which aspects of the school culture were healthier. Participation in the questionnaire was also used to help us understand the acceptability and feasibility of this approach.

#### 2.2.2 Staff interview and student focus group

To assess the acceptability and practicality of the processes and study design, interviews were conducted with key staff involved. Students for the focused discussion were recruited from within the school. Semi-structured topic guides were used for the interviews and focus groups and data was stored in NVivo14.

##### 2.2.2.1 Analysis

Thematic analysis was used to understand and explore the feasibility and acceptability of the study and barriers and facilitators to the implementation of the processes.

## 3 Results

The recruited school was a new school not yet at full student capacity; the main liaison was the school Head teacher and Head of Physical Education (PE). Due to the short length of the study, we did not expect to see major changes at the level of the school and its population rather we wanted to assess the feasibility and acceptability of the study design and capturing questionnaire data.

### 3.1 The Lifestyle and school questionnaire

The questionnaire was administered in the first month of the school year (September 2022), before the first audit; 237 students completed (95 % response rate). This was repeated at the end of the school year (June 2023), before the second audit was administered; with 210 (84 % response rate) responses. Repeating the questionnaire before the school implemented any changes gave us a sense of whether behaviours, feeling and attitudes changed over the course of the school year, in the absence of any intervention, as well as informing our assessment of the acceptability of this method.

At both time points the questions were well completed with ≥97 % of the respondents completing the physical activity, diet and school domain questions and 93 % of students completing the wellbeing domain, which included the largest number of questions. The survey scores were not normally distributed and are therefore reported as median and interquartile range (IQR).

**Fig. 2:**
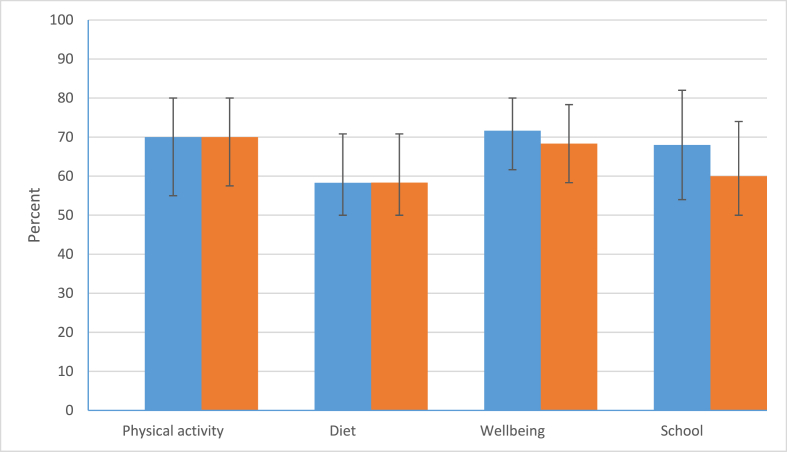
Median and interquartile range questionnaire results for the four domains for the first and second questionnaire responses (all responses).

At the time of the first questionnaire, the median percent for the physical activity domain was 70 % (IQR 55 %–80 %); diet was 58 % (IQR 50 %–71 %), wellbeing 72 % (IQR 62 %–80 %) and school 68 % (IQR 54 %–82 %). The questionnaire responses clearly identified diet as the priority for this school. The questionnaire results from the first and second administration are illustrated in Fig. 2. These clearly show that there was very little change in the physical activity and diet scores across the school year, but some reductions in wellbeing and school scores.

#### 3.1.1 Sensitivity analyses

We undertook two sensitivity analyses with the questionnaire data. Firstly, the questionnaire data had not been collected in a way to ensure that we had repeated measures from the same children. Subsequently, the differences in the responses could be due to changes in the sample. However, it was possible to match (based on name) around 130 (depending on the domain, approximate 50 % of the eligible population) responses. The matched results are shown in Appendix 3 and do not markedly differ from the unmatched results presented in this paper. Secondly, the final four questions asked specifically about the ease and importance of health, diet, activity and mental health at the school. We compared the physical activity, diet and wellbeing domain scores across the five Likert levels of agreement response options for each of these questions. This exploratory analysis found some indications of gradients in the responses with those reporting that being healthy was easier and more important also reporting healthier behaviours and attitudes, especially the wellbeing domain. These exploratory findings are reported in Appendix 4, as the four questions being separately analysed were part of the school domain, the question responses and school domain score is correlated and therefore we did not repeat this analysis for the school domain. These sensitivity analyses contribute to our understanding of the feasibility and validity of using the Lifestyle and School Questionnaire within the evaluation of a complexity-informed health promoting schools’ intervention.

### 3.2 The HPS process audit

**Fig. 3:**
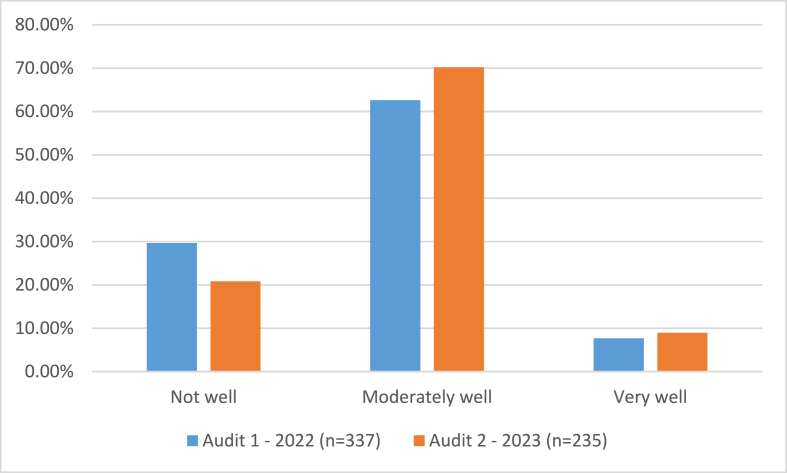
Comparison of student responses to audit question: How well does you school support you to eat and drink healthy foods.?.

The audit was circulated by the school in November 2022 and repeated November 2023. The results from the first audit reflect what we captured in the baseline questionnaire responses, 70.3 % students felt the school supported them ‘moderately’ or ‘well’ to eat and drink healthy options, while 29.7 % felt the school did not, see Fig. 3. 70.9 % students felt the school ‘did not’ or only ‘moderately’ supported them to be PA. 73.3 % students felt the school ‘did not’ or only ‘moderately’ supported them to look after their mental wellbeing. These students' responses were mirrored in the staff and parent responses.^4^ The school Head, the Head of PE and canteen manager were the staff who met to decide which areas highlighted by the audit the school could focus on given the resources available to them, which was procuring a new outside food service in September 2023 and the purchase of a physical activity app for students. Unlike the questionnaire follow-up data, the second audit showed slight improvement in student's satisfaction with the school food provision, 79.1 % felt the school ‘moderately’ or ‘well’ supported them to eat and drink healthy options and 20.7 % felt the school ‘did not’, see Fig. 3. 78.2 % students felt the school ‘did not’ or only ‘moderately’ supported them to be physically active. 82.1 % students felt the school ‘did not’ or only ‘moderately’ supported them to look after their mental wellbeing.

### 3.3 The process evaluation

Three key staff members (Head teacher, Head of PE and canteen manager) took part in an interview and a focused discussion was conducted with students from the school's pupil parliament (n = 30). The school we worked with was engaged in the process at the start; however, their resources were very limited and therefore they were only able to make one significant change in the canteen. Staff reported finding the HPS process useful for reflecting on their school context. The theme of resource came up repeatedly as limiting the ability to make significant changes to the school environment; resource referred to time, money and personnel. ‘*Just time … it's just time’* (Staff interview). Staff were keen to continue to make changes to the school environment, although they mentioned keeping it a priority is a challenge. ‘*We had lots of good chats about how we wanted to be better and then it just falls off the list, doesn't it?’* (Staff interview). However, despite having a school toolkit available to them, which highlighted available, free resources they reported they did not have sufficient time to explore these resources, ‘*I think it is time with someone leading … if we could get kind of food as a leadership responsibility and you get a good leader leading it well then it would do really well …. it needs to be the priority on their list, and they drive it … it wasn't a top priority for them, it wasn't linked to their performance management*.’ (Staff interview). Students reported that they found the questionnaire feasible and acceptable to complete. They seemed less aware of the HPS process over all although did recall completing the audit, their main response was that they did not feel they had a strong voice in making changes and had not been engaged in the new school food procurement process *‘the communication between the school and catering company isn't clear. And there's no direct way for us, as the Pupil Parliament, to talk to the catering company.’* (Pupil parliament).

## 4 Discussion

The aim of this study was two-fold; firstly, to determine the feasibility and acceptability of implementing the HPS process and to capture changes the school made because of the process. Secondly, to administer a low burden research questionnaire to understand its feasibility and acceptability and ascertain whether it can act as an evaluative research tool alongside the HPS process.

How much a school engages with the HP processes can vary and will depend on the Head teacher (principal), business manager and leadership teams' personal understanding of the importance of creating a HP culture within their school. It also depends on the resources a school has available to them however it is worth bearing in mind that within a complex system a small change can have a large effect and vice versa [24]. Whilst the HPS process has been designed to engage school staff, students and their families to co-create a response, the lack of time and financial resources restricted the school's response to the audit and limited a ‘whole school response’. The pupil parliament struggled to remember the process, suggesting the time taken to initiate a response resulted in the process having little impact on them. It is tempting to speculate that this could have played into the deterioration in the school culture questionnaire results; a more engaged approach from the school might have led to students feeling they have a voice and concerns were acted on.

The underlying theory of how we conceive the HPS process to create the conditions for healthy lifestyles (Fig. 1) suggests that whilst it is unlikely that schools would be able to implement significant change over one academic year, it is an iterative process of problem identification and response which creates culture change; hence as schools reach their short, middle and longer-term goals this would be reflected in changing priorities in future annual audits and the creation of a culture of health promotion.

How we capture complex system change in a school context is challenging and the extent to which questionnaires are a valid way to capture system level change needs consideration. In-depth methods, such as case studies, are advocated as a means of capturing system level change [25,26]; and these would provide a more comprehensive understanding of a dynamic school context, however they are time consuming to conduct and although useful for research purposes, they are not feasible for nationwide implementation. The HPS process was designed to help schools identify and respond to local barriers to health and, by repeating the process, understand the health trajectory of their school system [27]. A complex systems approach to capturing change explicitly brings a relational focus to the evaluation design, foregrounding the local and wider contexts. Our challenge was to develop a low burden questionnaire which captured signals of change in the culture or behaviour of the system by asking questions related to school culture, as well as any impact on student's health related behaviours. The questionnaire performed well in relation to being a reliable evaluative research tool as it picked up the same priority as the audit at the initial time point. Its validity needs further testing over a longer period and in other schools. To capture the impact of the HPS process we need to find ways to efficiently deliver the questionnaire and ideally in a way that means participants can be matched over time, bearing in mind the difficulties of collecting data in schools when you lose and gain a year group each academic year. There is also an inherent tension between capturing the impact of an intervention for research purposes and having an intervention, which is intended to impact school culture and pick up changes over time, hence the tension is two-fold, how one captures systems which are changing and how one does it in such a way that it is not burdensome for the school.

### 4.1 Limitations

System level change takes time; a limitation of this work was the misalignment of the school's timescales and the research process; ideally to capture system level change, the questionnaire would have been repeated after the implementation of a new canteen process, but this was considered unfeasible by the school. The Lifestyle and School Questionnaire only captured the perspective of adolescents and arguably, to capture whole school system change a questionnaire designed for school staff and families would show whether school staff, students and families, identified any change in culture.

Within our analysis of the questionnaire data, each question was given the same weighting within each of the four domains. While it may be possible that some questions warranted higher weightings than others did, the weightings are likely to vary between schools and samples as the health promoting focus of the schools differ. Therefore, equal weighting was appropriate for this exploratory study into efficient approaches to evaluating a complex intervention.

## 5 Conclusion

This study demonstrates the acceptability and feasibility of the HPS process; evaluating its impact needs future, longer-term research to ascertain if, once schools have made further changes and sustained current ones, the questionnaire would capture these changes over time and be mirrored in the school's annual audit. Further research on how to evaluate complex public health interventions that take a systems approach in schools, is needed to understand how we capture the complexities of system level change without burdening schools.

## Conflict of interest

Authors declare that there is no conflict of interest.

## Footnotes

1 Academies receive funding directly from the government and are run by an academy trust. They are inspected by Ofsted.

2 In England a Free School Meal (FSM) is a statutory benefit available to school aged children from families who receive other qualifying benefits.

3 Year 11 students (15–16 year olds were not involved at the request of the school due to this being their final year and a focus on exams).

4 Due to low responses from staff (*n = 22*) and parent/carers (*n = 49*) in first audit and the school not circulating the second audit to staff and parent/carers we have not included their response.
