# Supplementary material for: Feasibility study of the implementation of health promoting processes in a secondary school and ways to capture its impact on adolescent lifestyle choices
Source: Public Health Pract (Oxf). 2025 Feb 15;9:100591. doi: 10.1016/j.puhip.2025.100591 (PMC11891730; doi:10.1016/j.puhip.2025.100591)
Supplement: Multimedia component 1 [file mmc1.pdf]

HPS audit - student

1. Do you think it is important to have a healthy lifestyle? *

- [ ] No
- [ ] Yes

2. If you answered NO please explain why not in the box below:

3. How well do you think your school supports you to eat healthy foods and drinks? *

- [ ] Not well
- [ ] Moderately well
- [ ] Very well

4. What does your school do WELL support you to have a healthy diet? (please tick all that apply) *

- [ ] Availability of healthy foods and drinks in the canteen
- [ ] Availability of healthy foods and drinks in vending machines/snack areas
- [ ] Provides good access to drinking water
- [ ] Educates students in lessons on healthy eating and its benefits
- [ ] Provides guidance on what foods and drinks students can or can't bring into school
- [ ] Provides good role models
- [ ] Other

5. If you selected Other, please explain:

6. What does your school do LESS WELL to support you to have a healthy diet? (tick all that apply) *

- [ ] Does not provide enough healthy foods and snacks in the canteen
- [ ] Does not provide enough healthy foods and snacks in vending machines/snack areas
- [ ] Does not provide good access to drinking water
- [ ] Does not educate students in lessons on healthy eating and its benefits
- [ ] Does not provide guidance on what food and drink students can bring into school
- [ ] Does not provide good role models
- [ ] Other

7. If you selected Other, please specify:

8. What would you change in your school to improve your ability to have a healthy diet? *

9. How well do you think your school supports you to be as active as possible? *

- [ ] Not well
- [ ] Moderately well
- [ ] Very well

10. What does your school do WELL to support you to be active? (tick all that apply)

- [ ] Provides enough opportunities for students to be active in PE lessons
- [ ] Provides enough extra-curricular (outside of PE) opportunities for students to be active
- [ ] Provides students with a wide variety of active choices
- [ ] Educates students on the importance of being active

- [ ] Other

11. If you selected Other, please explain:

12. What does your school do LESS WELL to support you to be active?

- [ ] Does not provide enough opportunities for students to be active
- [ ] Does not provide enough extra-curricular (outside of PE) opportunities for students to be active
- [ ] Does not provide students with a wide variety of active choices
- [ ] Does not educate students on the importance of being active
- [ ] Other

13. If you selected Other, please specify:

14. What would you change in your school to help you be as active as possible? *

15. How WELL does your school support you to look after your mental wellbeing? *

- [ ] Not well
- [ ] Moderately well
- [ ] Very well

16. Are there ways that your school could better support your mental well being? *

Page 1

17. Is there anything else you think your school could do to create a healthy lifestyle's culture?
