# Supplementary material for: Feasibility study of the implementation of health promoting processes in a secondary school and ways to capture its impact on adolescent lifestyle choices
Source: Public Health Pract (Oxf). 2025 Feb 15;9:100591. doi: 10.1016/j.puhip.2025.100591 (PMC11891730; doi:10.1016/j.puhip.2025.100591)
Supplement: Multimedia component 2 [file mmc2.pdf]

Lifestyle and School Questionnaire 2022

Agreeing to take part

I have read and understood the information for the Health Promoting Schools project. I understand what I will have to do. My questions have been answered and I understand I can ask questions at any stage. ✱ *Required*

- Yes
- No

I understand that I do not have to take part and I am free to stop being part of the project without giving a reason. ✱ *Required*

- Yes
- No

I understand that once my name has been removed from the question form my responses will be used and cannot be removed from the research. ✱ *Required*

- Yes
- No

I understand that relevant sections of the data collected during the study, may be looked at by members of the research team. ✱ *Required*

- Yes
- No

I understand that no one will know my answers to the questions and they will be stored safely. ✱ *Required*

- Yes
- No

I understand that taking part should not harm me. ✱ *Required*

- Yes
- No

I understand that I can ask questions about the study at any time. ✱ *Required*

- Yes
- No

By clicking **next** you agree to take part in this online survey

Information about you

What is your name?

What year group are you in?

What is todays date?

Dates need to be in the format 'DD/MM/YYYY', for example 27/03/1980.

(dd/mm/yyyy)

Lifestyle and School Questionnaire (V1)

The following questions are about choices you make at school. There are four parts:

1. Questions on your activity
2. Questions on what you eat
3. Questions on what you feel
4. Questions about your school

Take your time to answer the questions, it should take about 20mins.

Questions about how active you are at school.

Over the past week, did you? (Tick the response that suits you best) *Optional*

	Never	Sometimes	Often	Always
Engage in energetic physical activity for 20 minutes for 3 days a week?	☐	☐	☐	☐
Exercise until your heart beat faster and you sweated?	☐	☐	☐	☐
Play active games with your friends?	☐	☐	☐	☐
Participate in school activities or sports after school hours?	☐	☐	☐	☐
Walk or do something active during your free time?	☐	☐	☐	☐

Questions about what you eat in school

Over the past week, did you? (Tick the response that suits you best)

	Never	Sometimes	Often	Always

Eat 2-4 portions of fruit each day	☐	☐	☐	☐
Eat 3-5 portions of vegetables each day	☐	☐	☐	☐
Choose low-fat milk or low-fat dairy foods	☐	☐	☐	☐
Drink six (6) or more glasses of water each day	☐	☐	☐	☐
Drink sweetened drinks such as fizzy, juice or squash?	☐	☐	☐	☐
Did you have breakfast before coming to school?	☐	☐	☐	☐
Did you avoid ‘‘sweets’’ or other foods high in sugar in school	☐	☐	☐	☐
Did you have school lunch?	☐	☐	☐	☐
Did you bring your own lunch to school?	☐	☐	☐	☐

Questions about how you feel at school

To what extent do the sentences below describe you? (Tick one answer for each statement)

	Not at all	A little	Somewhat	Quite a bit	A lot
I have people I look up to in school	☐	☐	☐	☐	☐
I work with people around me	☐	☐	☐	☐	☐
Getting an education is important to me	☐	☐	☐	☐	☐

I know how to behave in different social situations	☐	☐	☐	☐	☐
People think that I am fun to be with	☐	☐	☐	☐	☐
I am able to solve problems without harming myself or others (for example by using drugs and/or being violent)	☐	☐	☐	☐	☐
I feel supported by my friends	☐	☐	☐	☐	☐
I know where to go in my community to get help	☐	☐	☐	☐	☐
I feel I belong at my school	☐	☐	☐	☐	☐
I have opportunities to show others that I am becoming an adult and can act responsibly	☐	☐	☐	☐	☐
I am aware of my own strengths	☐	☐	☐	☐	☐
I feel safe when I am in school	☐	☐	☐	☐	☐
I have opportunities to develop skills that will be useful later in life (like job skills and skills to care for others)	☐	☐	☐	☐	☐

Questions about your school

To what extent do the sentences below describe your school? (tick one answer for each statement)

	Not at all	A little	Somewhat	Quite a bit	A lot
There's at least one teacher or other adult in this school I can talk to if I have a problem	☐	☐	☐	☐	☐
In this school, students' ideas are listened to and valued	☐	☐	☐	☐	☐
This school really cares about students as individuals	☐	☐	☐	☐	☐
At this school, students have a lot of chances to help decide and plan things like school activities, events and policies	☐	☐	☐	☐	☐
Student activities at this school offer something for everyone	☐	☐	☐	☐	☐

Students at this school are encouraged to take part in activities, programs and special events	☐	☐	☐	☐	☐
This school cares about students health	☐	☐	☐	☐	☐
At this school it is easy to make healthy food choices	☐	☐	☐	☐	☐
At this school it is easy to be active	☐	☐	☐	☐	☐
At this school it is easy to look after my mental health	☐	☐	☐	☐	☐

Final page

Thank you for completeing this survey, your answers are so valuable.
