# Supplementary material for: Feasibility study of the implementation of health promoting processes in a secondary school and ways to capture its impact on adolescent lifestyle choices
Source: Public Health Pract (Oxf). 2025 Feb 15;9:100591. doi: 10.1016/j.puhip.2025.100591 (PMC11891730; doi:10.1016/j.puhip.2025.100591)

Feasibility study of the implementation of health promoting processes in a secondary school and ways to capture its impact on adolescent lifestyle choices

Camilla Forbes, Andrew James Williams and Katrina Wyatt

Appendix 3: Sensitivity analysis using only those pupils who questionnaire responses could be matched from the first to second sweep of the questionnaire

The data are median and interquartile range

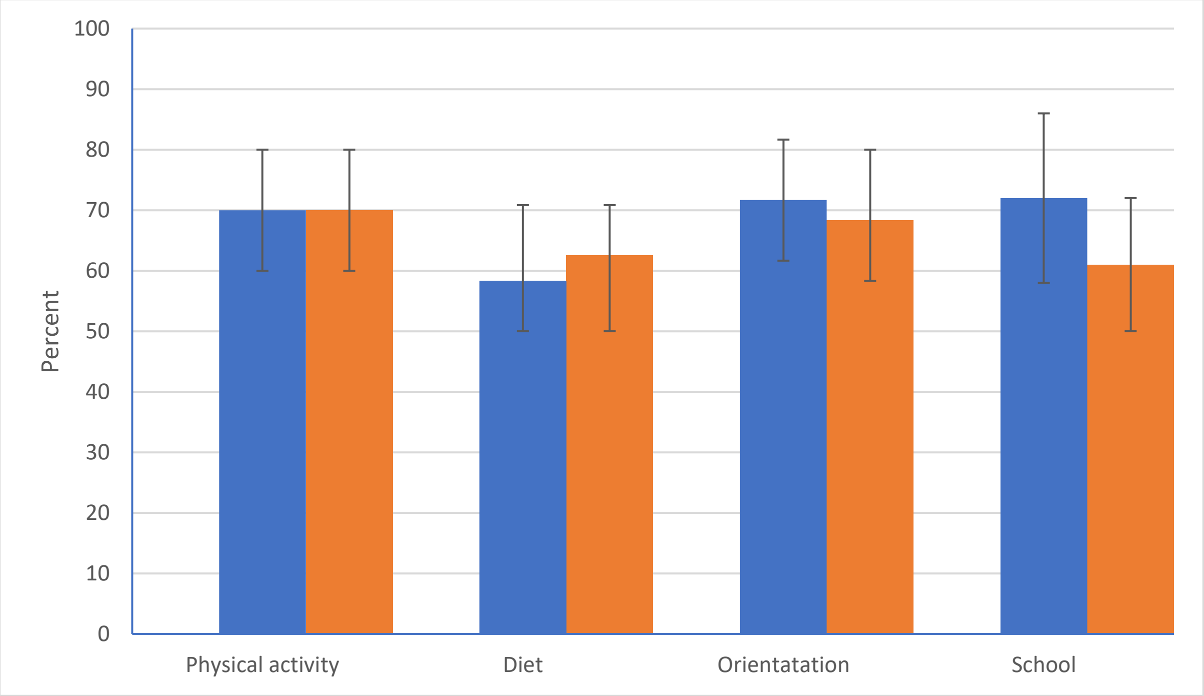

Supplement: Multimedia component 3 [file mmc3.pdf]
