# Supplementary material for: Feasibility study of the implementation of health promoting processes in a secondary school and ways to capture its impact on adolescent lifestyle choices
Source: Public Health Pract (Oxf). 2025 Feb 15;9:100591. doi: 10.1016/j.puhip.2025.100591 (PMC11891730; doi:10.1016/j.puhip.2025.100591)
Supplement: Multimedia component 4 [file mmc4.pdf]

Feasibility study of the implementation of health promoting processes in a secondary school and ways to capture its impact on adolescent lifestyle choices

Camilla Forbes, Andrew James Williams and Katrina Wyatt

Appendix 4: Sensitivity analysis exploring the responses to the physical activity, diet and wellbeing domain responses to key questions on the ease and importance of health in the school

The data are median and interquartile range

Question: This school cares about student health

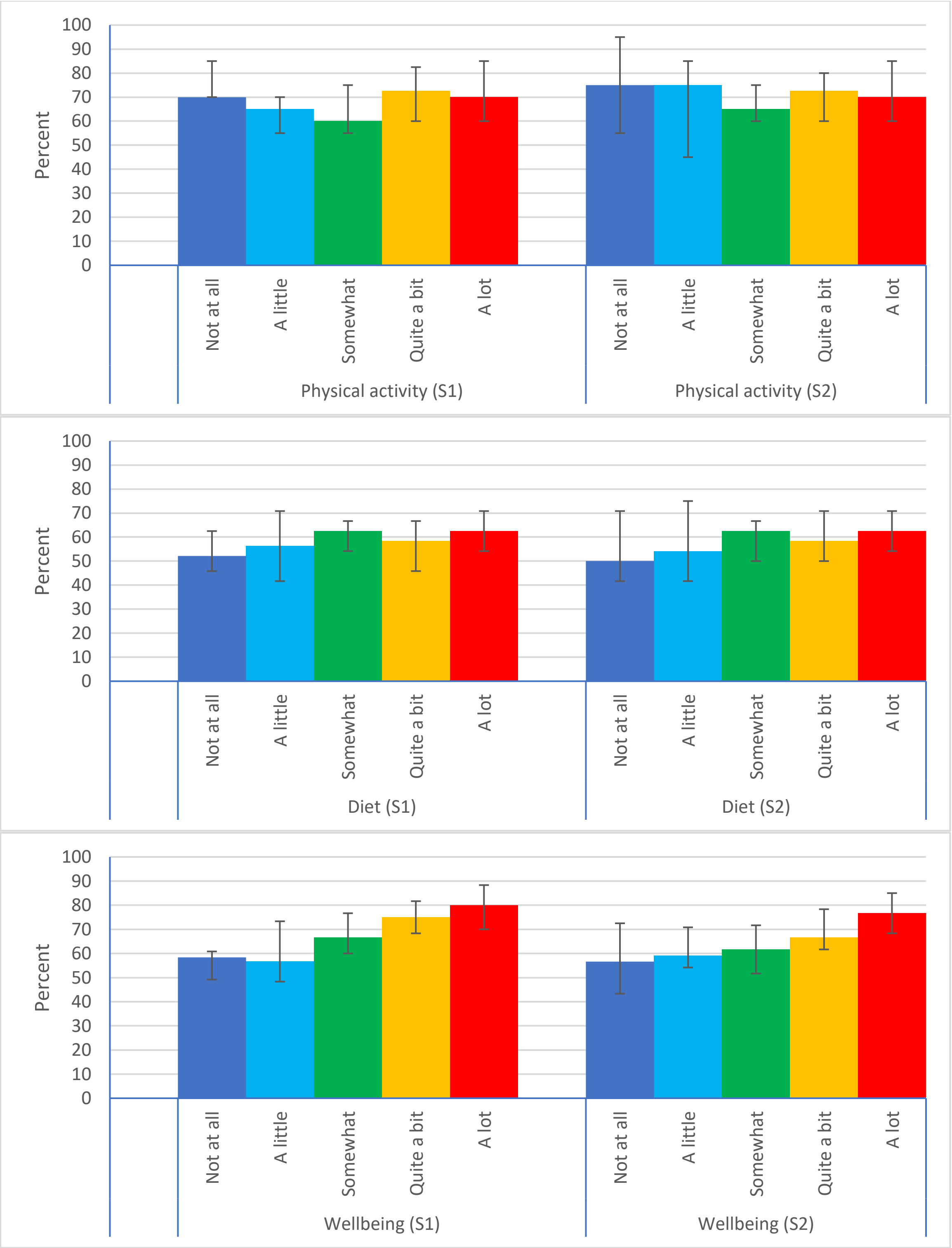

S1: is the first sweep of questionnaire collection at the start of the school year, S2 is the second sweep at the end of the school year

Feasibility study of the implementation of health promoting processes in a secondary school and ways to capture its impact on adolescent lifestyle choices

Camilla Forbes, Andrew James Williams and Katrina Wyatt

Question: At this school is it easy to make healthy food choices

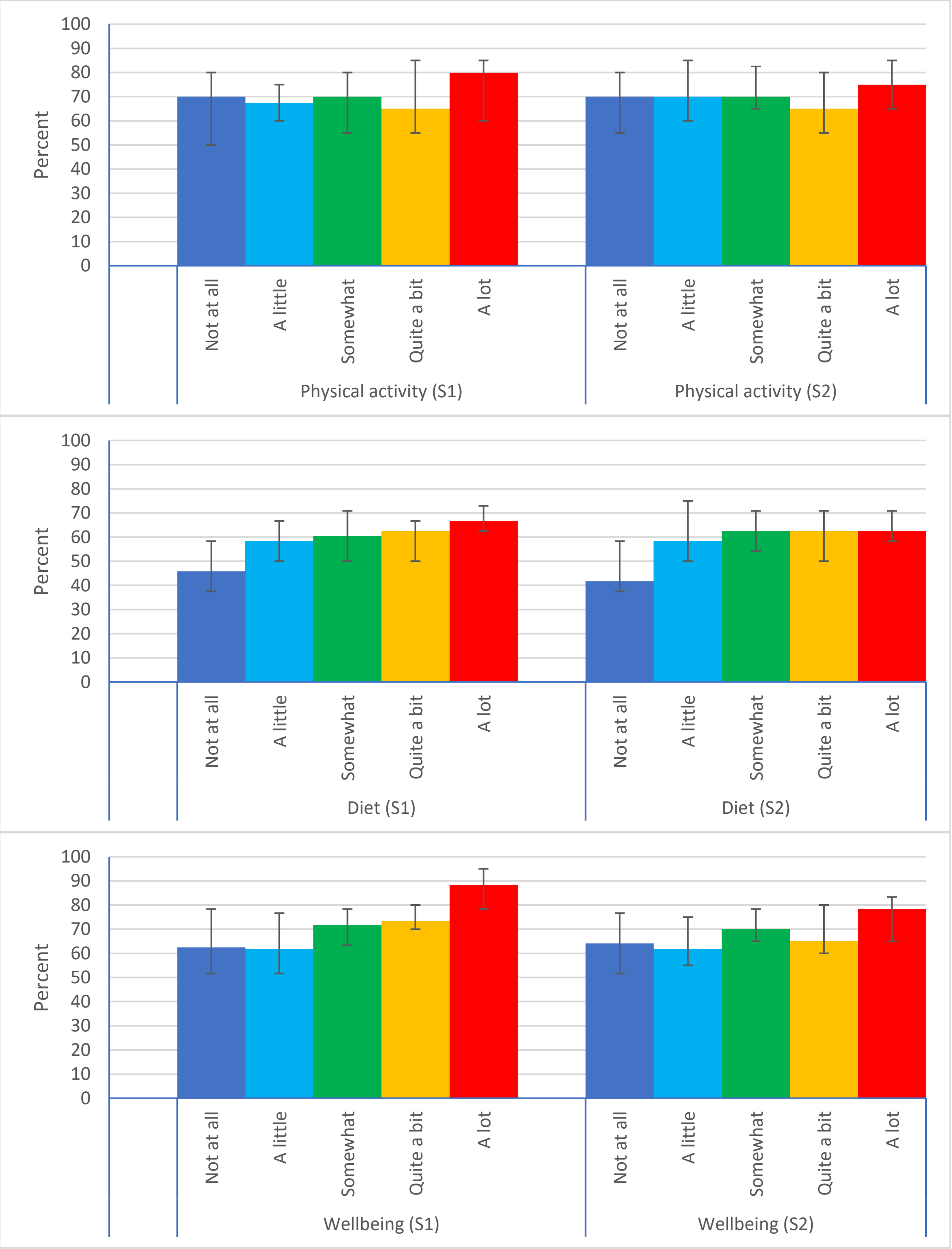

S1: is the first sweep of questionnaire collection at the start of the school year, S2 is the second sweep at the end of the school year

Feasibility study of the implementation of health promoting processes in a secondary school and ways to capture its impact on adolescent lifestyle choices

Camilla Forbes, Andrew James Williams and Katrina Wyatt

Question: At this school it is easy to be active

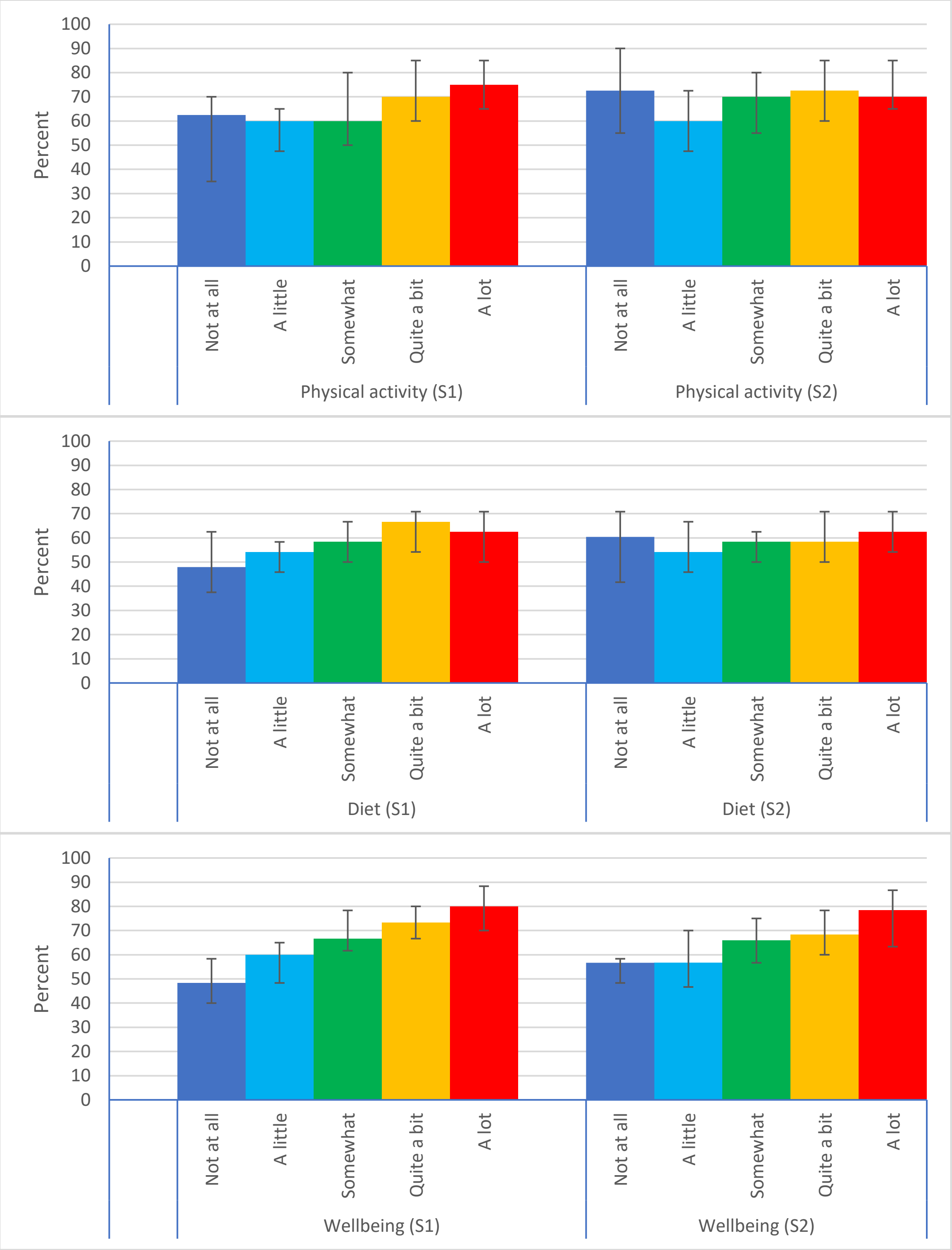

S1: is the first sweep of questionnaire collection at the start of the school year, S2 is the second sweep at the end of the school year

Feasibility study of the implementation of health promoting processes in a secondary school and ways to capture its impact on adolescent lifestyle choices

Camilla Forbes, Andrew James Williams and Katrina Wyatt

Question: At this school it is easy to look after my mental health

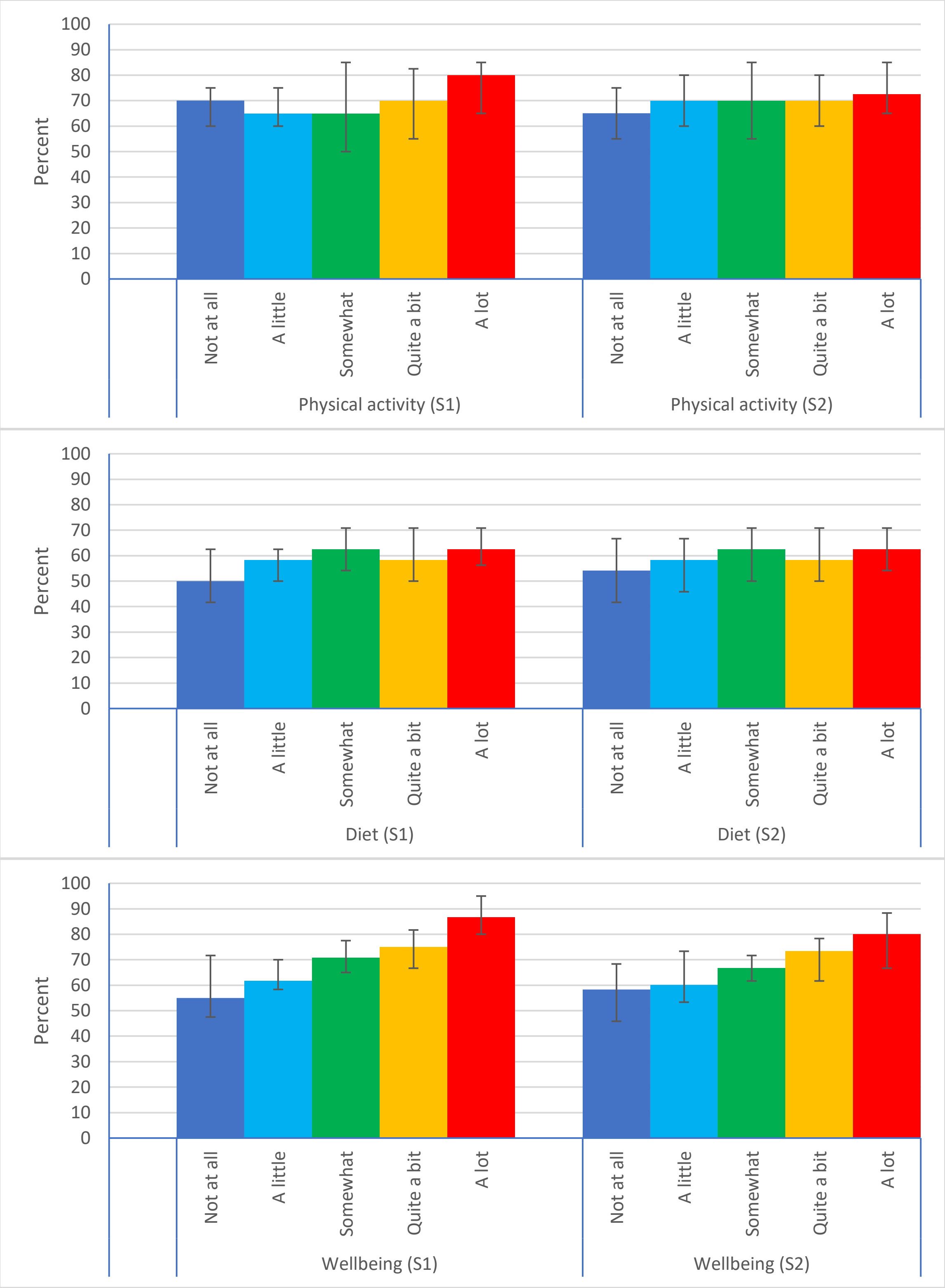

S1: is the first sweep of questionnaire collection at the start of the school year, S2 is the second sweep at the end of the school year
